# B-cell receptor signaling studies in patients with lupus: preliminary results

**DOI:** 10.1186/ar3994

**Published:** 2012-09-27

**Authors:** M Faludi, A Mao, E Vinet, A Clarke, C Pineau, S Bernatsky, E Nashi

**Affiliations:** 1 McGill University, Montreal, QC, Canada; 2 McGill University Health Centre, Montreal, QC, Canada

## Background

There is significant evidence from murine and human genomic studies that B-cell receptor (BCR) signaling abnormalities are potential factors in the pathogenesis of lupus. However, data on signaling deviations in lupus patients are scant. We have undertaken a project to comprehensively study BCR signaling deviations in lupus patients.

## Methods

Peripheral blood mononuclear cells will be isolated and frozen. B cells will be stimulated with F(ab')2 anti-IgM and anti-IgG. Using eight-parameter flow cytometry, we will determine signaling amplitude, as measured by phosphorylated ERK1/2, in IgG memory cells (CD20^+^IgG^+^), mature naïve (CD20^+^,CD27^low^,CD38^low^), transitional (CD20^+^,CD38^high^,CD10^high^), B1 (CD20^+^,CD27^high^) and IgM memory B cells (CD20^+^,IgM^+^,CD27^high^,CD86^high^). We will measure pERK levels at baseline (time 0), 1 minute (early signal), 5 minutes (peak signal) and 15 minutes (late signal). We will establish normal parameters by studying BCR signaling in 100 nonautoimmune individuals. We will then determine the prevalence of low and high BCR signaling deviations in 100 lupus patients. Signaling deviations will be correlated with clinical data.

## Preliminary results

**Figure 1:**
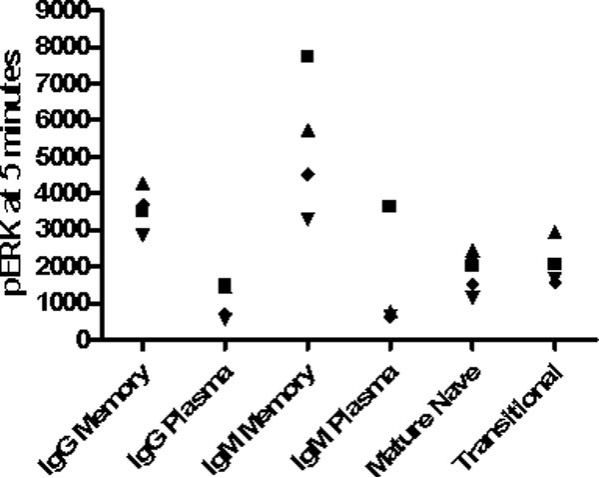


We have optimized a protocol that enables us to identify each of the above B-cell subsets while assaying phospho-ERK levels. Results from four lupus patients indicate that this protocol is able to identify signaling differences between individuals with lupus. See Figure 1.

## Conclusion

The extent to which signaling deviations contribute to autoimmunity in patients with lupus remains to be determined. By elaborating a robust method to assay BCR signaling, we hope not only to measure this pathogenic factor but also to answer fundamental questions, such as whether diminished BCR signaling correlates with a broader autoimmune phenotype, as would be postulated by the impaired negative selection that is caused by diminished signaling. In the future, we will aim to convert signaling studies from research tools to clinical tools.

